# The effect of patient-prosthesis mismatch on survival after aortic and mitral valve replacement: a 10 year, single institution experience

**DOI:** 10.1186/s13019-019-1034-4

**Published:** 2019-12-06

**Authors:** Sudeep Das De, Ashok Nanjappa, Karim Morcos, Sadia Aftab, John Butler, Vivek Pathi, Philip Curry, Sukumaran Nair

**Affiliations:** Department of Cardiothoracic Surgery, Golden Jubilee National Hospital, Glasgow, UK

**Keywords:** Aortic valve, Mitral valve, Survival

## Abstract

**Background:**

The evidence on the impact of patient-prosthesis Mismatch (PPM) on survival thus far has been conflicting. The aim of this study was to 1) study the effect of PPM on survival after isolated aortic and mitral valve replacement and 2) Assess the interaction between left ventricular function and PPM on survival.

**Methods:**

The study cohort was patients who underwent isolated Aortic valve replacement (AVR) and Mitral valve replacement (MVR) over a 10-year period from 2008 to 2018. PPM was defined using the projected indexed effective orifice area (EOAi). The cohort was divided into different groups based on the degree of PPM. The severity of PPM was classified using threshold values of EOAi used in the literature. The Kaplan- Meier method was used to compare survival by degree of PPM. Multivariate Cox proportional hazards models were used to generate adjusted hazard ratios (HR) with 95% confidence intervals. An interactive term for ejection fraction (EF) was added to test whether EF modifies the effect of the PPM grade on survival. In addition, sub-group analysis based on left ventricular function was performed.

**Results:**

In the AVR cohort, there were a total of 1953 patients. The distribution of patients in the different PPM categories was as follows: no PPM 59.7%; moderate PPM 36.8%; severe PPM 3.5%. There was no significant difference in survival between the different groups. At 10 years, the adjusted HR between patients with severe PPM versus no PPM was 1.1(CI 0.5–2.4, *p* >  0.05) and the HR between those with moderate PPM versus no PPM was 0.97 (CI 0.74–1.23, *p* >  0.05). In the MVR cohort, there were a total of 298 patients. The distribution of PPM is as follows: no PPM 59.4%; and with PPM 40.6%. Again, there was no significant difference in survival between the groups. At 5 years, the adjusted HR between patients with PPM versus no PPM was 1.45 (CI 0.67–3.14, *p* >  0.05). In both groups, there was no significant interaction between left ventricular function (LVF) and degree of PPM on survival.

**Conclusions:**

In our study cohort, the degree of PPM was not an independent predictor of survival after AVR or MVR. There was also no significant interaction between LV function and degree of PPM on survival.

## Background

The concept of patient-prosthetic mismatch (PPM) was first described by Rahimtoola et al. in 1978 [1]. In the aortic position, it occurs when there is an excessive trans-valvular gradient across a normally functioning prosthetic valve to generate an adequate cardiac output. Since then there have been numerous publications describing the prevalence and clinical consequences of PPM. The evidence thus far has been conflicting with several studies reporting higher mortality and increased incidence of re-operations associated with PPM [2–7], whereas others have shown no clinical relevance of PPM [8–13].The postulated harmful effects of PPM may be attributed to less left ventricular (LV) mass regression with persistent LV hypertrophy, diastolic dysfunction and subsequent systolic LV dysfunction. PPM in the mitral position occurs when there is residual mitral stenosis with higher trans-mitral gradients after mitral valve replacement. Mitral PPM has been less widely studied with recent publications revealing conflicting results [14–16]. In the mitral position, the potential effect on survival may be a result of increased left atrial pressure and pulmonary hypertension with subsequent right ventricular (RV) dysfunction.

The objective of this study was to 1) study the effect of Patient-prosthetic mismatch (PPM) on survival after isolated aortic and mitral valve replacement and 2) Assess the interaction between left ventricular function and PPM on survival.

## Methods

### Study cohort

We retrospectively analysed patients who underwent aortic valve replacement (AVR) and mitral valve replacement (MVR) at our institution over a 10-year period from 2008 to 2018. Data was extracted from our local database and included patient and operative variables which could be potential confounders. Several exclusion criteria which could potentially increase the risk profile of the patients and make our study cohort heterogenous were applied. These included patients who underwent concomitant coronary artery bypass grafting, infective endocarditis, emergency cases, Re-operations, additional aortic procedures (aortoplasty or aortic root replacements), aortic dissection and thoracic aortic aneurysm repair operations. The patients were stratified based on the degree of PPM.

### Definition of PPM

Data was collected on the valve prosthesis model type, size and the patients’ height and weight. The effective orifice area (EOA) of each prosthesis model type and size was obtained from the literature and company websites. The effective orifice area index (EOAi) was obtained by dividing the EOA by the patient’s body surface area (BSA). PPM was defined using the value of the EOAi; this has been the measure widely accepted and validated in the literature. The severity of PPM was classified using threshold values of EOAi used in the literature.

The patients who underwent AVR were divided into 3 groups based on the severity of PPM: Group 1: No PPM (EOAi >/Patient characteristics 0.85 cm^2^/m^2^), Group 2: Moderate PPM (0.65 < EOAi < 0.85 cm^2^/m^2^) and Group 3: Severe PPM (EOAi =/< 0.65 cm^2^/m^2^). The MVR patients were divided into 2 groups: Group 1: No PPM (EOAi >/= 1.2 cm^2^/m^2^), Group 2: PPM present (EOAi < 1.2 cm^2^/m^2^).

### Outcome measures

The primary outcome measure was survival based on the degree of PPM. Ejection fraction (EF) was used as the measure of left ventricular function. Subgroup analysis was performed based on the ejection fraction.

### Statistical analysis

Continuous data was expressed as mean with a standard deviation (SD) or median with an interquartile range (IQR). Categorical variables were expressed as a percentage. Baseline characteristics and operative variables across the strata of PPM were compared with Student’s 2 sample T-test or the Man-U Whitney test for continuous variables and the chi2 squared test or Fischer’s exact test for categorical variables. The Kaplan- Meier method was used to compare survival by PPM level. The log rank test was used to detect a difference among the different levels. Multivariate Cox proportional hazards models were used for mortality to generate adjusted hazard ratios (HR) with 95% confidence intervals. The hazard ratio was adjusted for the following covariates: Age, Gender, Body mass index (BMI), Logistic EuroSCORE, history of Diabetes Mellitus (DM), Hypertension, Peripheral vascular disease (PVD), Cross clamp time, pre-op Atrial fibrillation (AF) and need for post-op permanent pacemaker. An interactive term for ejection fraction (EF) was added to test whether EF modifies the effect of the PPM grade and survival. In addition, sub-group analysis based on ejection fraction was performed. All statistical analysis was performed using Stata version 12. For all statistical analyses, data are presented with 95% confidence intervals and a *p* value < 0.05 was considered statistically significant.

## Results

### Patient characteristics and operative data

There were a total of 1953 patients who underwent isolated AVR in our cohort. The median follow-up time was 6.3 years. The mean age of the patients was 68.5 years and 53.7% were males, with a mean BMI was 29.1 kg/m2. Overall 16.5% of patients (*n* = 323) had an EF of less than 50% and the median logistic EuroSCORE was 4.92. The distribution of patients in the different PPM categories is as follows: no PPM 59.7% (*n* = 1166); moderate PPM 36.8% (*n* = 719); severe PPM 3.5% (*n* = 68). Patients with severe PPM were more frequently male (63.2%), had a higher BMI (mean 35.4), and had a higher percentage of diabetic (39.7%) and hypertensive (85.3%) patients.

There were a total of 298 patients in the isolated MVR cohort. The median follow-up time was 7.1 years. The mean age of the patients was 62.5 years and 39.3% were males with a mean BMI of 26.5 kg/m2. There were 17.4% of patients (*n* = 52) with an EF < 50% and the median Logistic EuroSCORE was 5.1. The distribution of PPM is as follows: no PPM 59.4% (*n* = 177); and with PPM 40.6% (*n* = 121). In the MVR group, patients with PPM were older (mean age 67 years) and had a higher Logistic EuroSCORE (median 5.8). The details of the patient demographics and operative variables for AVR and MVR patients stratified by degree of PPM are presented in Tables 1 and 2 respectively.

**Table 1:** Patients’ (AVR) baseline characteristics and operative variables by degree of PPM

Patient Characteristics (AVR)	Overall *N* = 1953	No PPM EOAi> = 0.85 *N* = 1166	Moderate PPM 0.65 < EOAi< 0.85 *N* = 719	Severe PPM EOAi = < 0.65 *N* = 68	*P* value
Age, years (SD)	68.5(12.5)	67.1(13.8)	70.7(10)	68.9(8.7)	< 0.01
Gender/Male (%)	1048(53.7%)	650(55.8%)	355(49.4%)	43(63.2%)	< 0.01
BMI, kg/m2 (SD)	29.1(5.8)	27.6(5.3)	31.1(5.5)	35.4(6.4)	< 0.01
History of DM (%)	367(18.8%)	174(14.9%)	166(23.1%)	27(39.7%)	< 0.01
History of Hypertension (%)	1233(63.1%)	672(57.6%)	503(70%)	58(85.3%)	< 0.01
History of PVD (%)	182(9.3%)	98(8.4%)	74(10.3%)	10(14.7%)	> 0.05
Log EuroSCORE (IQR)	4.92(2.86–8.15)	4.81(2.71–8.1)	5.13(3.07–8.44)	4.25(2.44–6.76)	> 0.05
Cross Clamp time(IQR)	69(60–81)	69(60–81)	68(59–81)	71(65–90)	> 0.05
Pre-op AF (%)	259(13.8%)	153(13.1%)	100(13.9%)	6(8.8%)	> 0.05
Ejection Fraction < 50% (%)	323(16.5%)	221(19%)	93(12.9%)	9(13.2%)	< 0.01
Need for post-op Permanent Pacemaker (%)	26(1.33%)	20(1.72%)	6(0.83%)	0(0%)	> 0.05
Biological valve (%)	1601(82%)	888(76.2%)	651(90.5%)	62(91.2%)	< 0.01
NYHA					< 0.01
I	149 (11.1%)	99 (13.2%)	48 (8.9%)	2 (4%)	
II	644 (48.1%)	361 (48.2%)	254 (47.1%)	29 (58%)	
III	497 (37.1%)	262 (35.0%)	219 (40.6%)	16 (32%)	
IV	48 (3.6%)	27 (3.6%)	18 (3.3%)	3 (6%)

**Table 2:** Patients’ (MVR) baseline characteristics and operative variables by degree of PPM

Patient characteristics (MVR)	Overall *N* = 298	No PPM EOAi> = 1.2 N = 177	PPM EOAi< 1.2 N = 121	P Value
Age, years (SD)	62.5(13.5)	59.4(12.8)	67(13.3)	< 0.05
Gender/Male (%)	117(39.3%)	68(38.4%)	49(40.5%)	> 0.05
BMI, kg/m2 (IQR)	26.5(14.7–44.3)	26.3(23–30)	26.9(24.1–31.6)	> 0.05
History of DM (%)	21 (7%)	11 (6.2%)	10(8.3%)	> 0.05
History of Hypertension (%)	134(45%)	70(39.5%)	64(52.9%)	< 0.05
History of PVD (%)	16(5.4%)	8(4.5%)	8(6.6%)	> 0.05
Log EuroSCORE (IQR)	5.1(2.9–8.5)	4.5(2.4–7.5)	5.8(3.4–9.3)	< 0.05
Cross Clamp time(IQR)	73(59–91)	73(56–89)	73(60–97)	> 0.05
Pre-op AF (%)	135(45.3%)	77(43.5%)	58(47.9%)	> 0.05
Ejection Fraction < 50% (%)	52(17.4%)	34(19.2%)	18(14.9%)	> 0.05
Need for post-op Permanent Pacemaker (%)	8(2.7%)	5(2.8%)	3(2.5%)	> 0.05
Biological valve (%)	123(41.3%)	41(23.2%)	82(67.8%)	< 0.05
NYHA				> 0.05
I	27(9%)	17(9.6%)	10(8.3%)	
II	110(36.9%)	67(37.9%)	43(35.5%)	
III	128(43%)	70(39.6%)	58(47.9%)	
IV	33(11%)	23(13%)	10(8.3%)	

### Primary outcomes

**Fig. 1:**
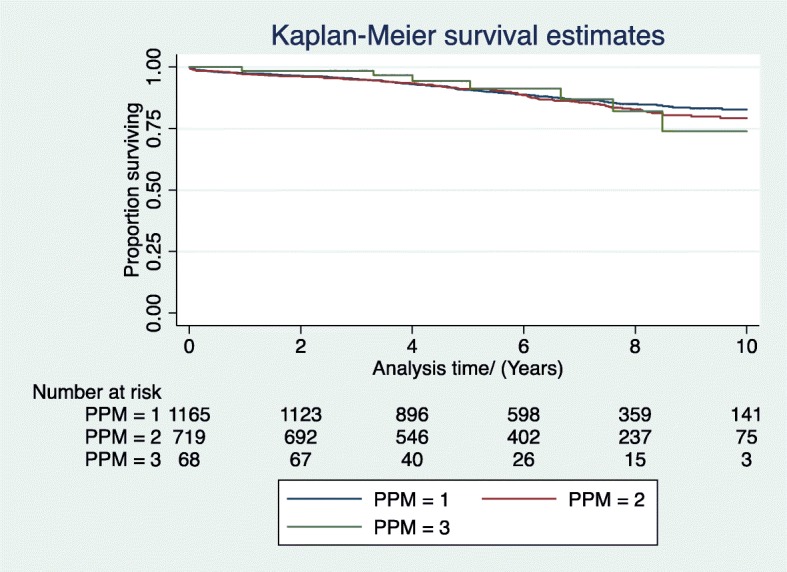
Unadjusted survival by degree patient-prosthesis mismatch (PPM); PPM = 1, No PPM, PPM = 2, Mod PPM, PPM = 3, Severe PPM)

In patients who underwent isolated AVR, there was no difference in survival between the different grades of PPM at 1, 5 and 10 years in unadjusted and adjusted analyses (Log rank test *p* > 0.05) (Fig. 1). There was also no significant trend in worsening survival based on grade of PPM. At 1 year, the adjusted hazard ratio (HR) between Group 3 (severe PPM) versus Group 1 (no PPM) was 0.71(CI 0.09–5.52, *p* > 0.05) and the HR between Group 2 (moderate PPM) versus Group 1 was 1.2 (CI 0.65–2.17, p > 0.05). After 10 years, the adjusted HR between Group 3 versus Group 1 was 1.1(CI 0.5–2.4, p > 0.05) and the HR between Group 2 versus Group 1 was 0.97 (CI 0.74–1.23, p > 0.05).

**Fig. 2:**
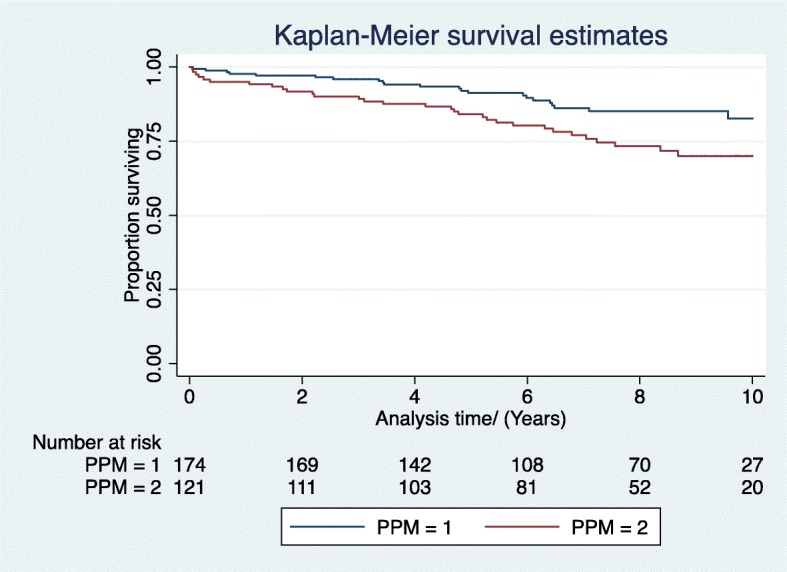
Unadjusted survival by degree patient-prosthesis mismatch (PPM); PPM = 1 No PPM, PPM = 2 PPM)

For the MVR cohort, there was a difference in survival between patients with PPM versus patients with no PPM at 1, 5 and 10 years in the unadjusted survival analysis (Log rank test *p* < 0.05) (Fig. 2). However, after adjustment, there was no survival difference between the groups. At 1 year, the adjusted HR between Group 2 (PPM) versus Group 1(No PPM) was 2.0 (CI 0.45–8.80, *p* > 0.05), at 5 years the HR between Group 2 versus Group 1 was 1.45 (CI 0.67–3.14, p > 0.05) and at 10 years, the HR between Group 2 versus Group 1 was 1.7 (CI 0.9–3.1).

### Subgroup analysis

**Fig. 3:**
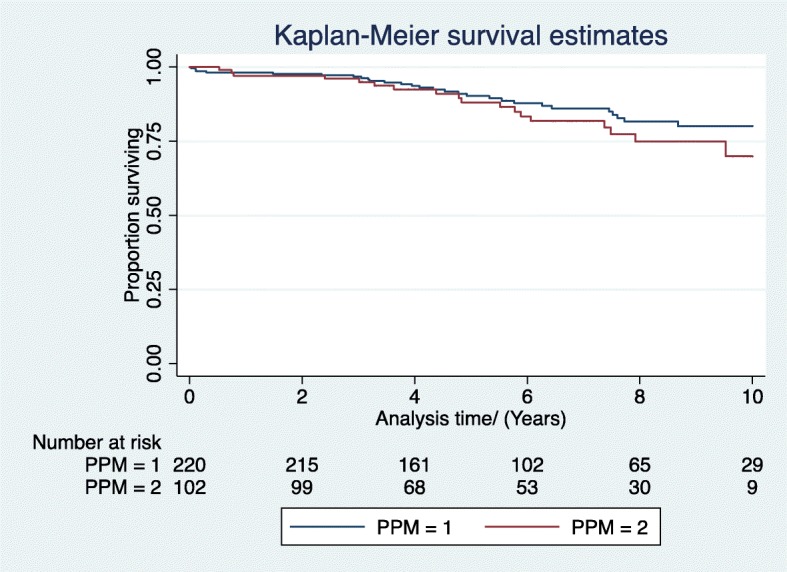
Subgroup Analysis- Survival curves Comparing Group1 (no PPM, EOAi > 0.85) vs Group2 (PPM, EOAi < 0.85) in patients with moderate and poor LV (EF < 50%)

Subgroup analysis was performed on patients who had an EF less than 50%. The cohort of patient who underwent AVR was dichotomized into patients without PPM (Group 1: EOAi > 0.85 cm2/m2) and with PPM (Group 2: EOAi < 0.85 cm2/m2). There was no difference in survival at 1,5 and 10 years in both adjusted and unadjusted analyses (Log rank test *p* > 0.05) (Fig. 3). At 1 year the adjusted HR between Group 2 versus Group 1 was 1.4 (CI 0.3–7.2, p > 0.05), at 5 years the HR was 0.8 (CI 0.4–2.0, p > 0.05) and at 10 years the HR was 1.2 (CI 0.6–2.3, p > 0.05). Multivariate analysis showed no significance for interactive term between PPM and LVF.

**Fig. 4:**
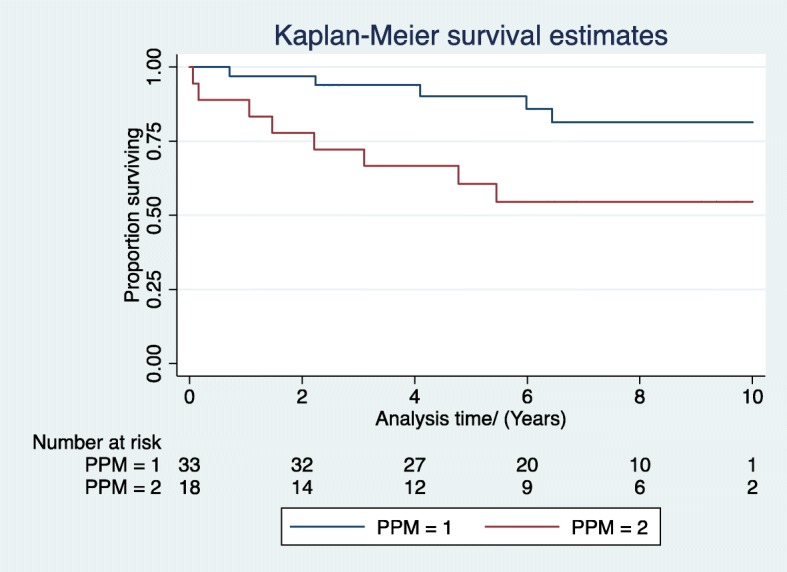
Subgroup Analysis- Unadjusted Survival curves Comparing Group1 (no PPM, EOAi > 1.2) vs Group2 (PPM, EOAi < 1.2) in patients with moderate and poor LV (EF < 50%)

In the MVR patients, subgroup analysis of patients with an EF < 50%, unadjusted analysis showed a significant difference in survival at 5 and 10 years between Group 2 (PPM) versus Group 1 (no PPM) (Fig. 4) However, after adjustment there was no difference in survival. At 5 years, the adjusted HR between Group 2 versus Group 1 was 3.5 (CI 0.85–14.6, *p* > 0.05) and at 10 years, the adjusted HR between Group 2 versus Group 1 was 4.0 (CI 0.98–16.6). Again, multivariate analysis showed no significance for interactive term between PPM and LVF.

## Discussion

The main findings in our study are as follows: 1) Amongst the cohort of patients who underwent isolated AVR, there was no significant association between degree of PPM and survival. 2) Amongst the MVR cohort, unadjusted analyses showed a significant improvement in survival in patients with no PPM. However, after adjustment, there was no survival benefit in patients without PPM. There was no significant interaction between left ventricular function/ejection fraction and PPM on survival.

These findings suggest that PPM is not an independent predictor of survival. This is consistent with previous studies [17, 18] that also showed that PPM is associated with a higher surgical risk profile and is a proxy for worse outcomes rather than being the cause. The decrease in late survival previously noted may have actually been due to this higher risk profile rather than the degree of PPM.

In addition, in our study cohort the mean age of the AVR cohort was 68.5 years and that in the MVR cohort was 62.5 years. Recent publications have revealed that the impact of PPM on survival is accentuated in a younger patient population [18]. Our study cohort was heterogeneous with patients who underwent AVR and MVR with biological and mechanical valves. The findings in our study could have been confounded by different haemodynamic profiles associated biological and mechanical valves as well as the bleeding or thrombotic complications associated with anticoagulation in patients who underwent mechanical valve replacement. A further source of heterogeneity in our study cohort was there were patients with stenosis, regurgitation and mixed disease. It has been hypothesized that the improved survival after AVR can be attributed to a lower trans-valvular gradient and subsequent reverse remodelling of the hypertrophied left ventricle (LV). In patients with aortic stenosis (AS), primarily there is concentric hypertrophy of the LV, whereas in aortic regurgitation (AR), the left ventricle is primarily dilated. Previous studies [19] have shown that the pathophysiology of reverse remodelling is different after valve replacement in patients with predominantly aortic stenosis versus aortic regurgitation and this might have affected long term survival and confounded our findings.

In this study, PPM was defined using the projected effective orifice area (EOA) derived from the literature and industry generated charts; this has been widely accepted as an accurate marker to grade PPM. A recent paper by Amorim et al. [20] questioned the validity of using projected EOA in different study populations. The main reason for this is that EOA is a patient specific parameter; the individual patient’s flow velocity across the valve and the geometry of the left ventricular outflow tract needs to be taken into account to calculate an in-vivo EOA post valve implantation. In addition to that, in-vivo trans-valvular gradients can be used to correlate to the degree of PPM. The conflicting data in the literature regarding PPM could be explained potentially by the misclassification of PPM using projected EOA as a marker.

In the subgroup analysis, there could be several reasons to explain the finding that there was no interaction between left ventricular function and degree of PPM on survival. As the number of patients in our cohort with impaired left ventricular function (EF < 50%) was low, this study may have been underpowered to detect any significant difference. In patients with a reduced EF, the stroke volume is low which might in turn not generate a significant trans-valvular gradient. Therefore, the low predicted EOAi in patients with moderate and severe PPM may be enough to result in reverse remodelling and there is no added effect of EF on survival in patients with PPM. A similar finding was observed in patients with low gradient AS where PPM had no impact on survival [21].

Strategies to prevent PPM include pre-operative calculation of the projected EOAi and appropriate sizing, the use of stentless valves, and securing valves in the supra-annular position. Aortic root enlargement procedures can also be performed; however, the risk of these procedures has to be weighed against the evidence presented here and in previous publications where there is no conclusive increase in mortality associated with PPM, especially in an older patient population. Although surgical techniques have been described in the literature, to implant a larger prosthesis in the mitral position and thereby improve EOAi, these techniques are not always simple and without potential risks of complications. Particularly with the lack of prognostic benefit following such complex techniques, one has to carefully weigh the benefits against the risks involved in attempts to implant larger mitral prosthesis in an attempt to abolish PPM in aortic and mitral valve replacement.

## Limitations

There are several limitations in our study. Firstly, this was a retrospective study. There could have been residual confounding even after adjustment for multiple covariates. The number of patients was low especially in the severe PPM category in the AVR group and in the MVR cohort; the study could therefore have been underpowered to detect any significant association. The generalisability of the study to a wider patient population can be questioned given that there was a selection bias towards older patients in our cohort. We did not have data on echocardiography in the medium or long term after surgery; it would have been useful to correlate the degree of PPM to long term hemodynamic data. From our database, we had information on the survival status of the patients, but not the cause of death; hence it was not possible to differentiate between cardiovascular and non-cardiovascular causes of death. We also did not have data on the long-term quality of life of our patients; it would have been informative to know if PPM had an impact quality of life.

## Conclusions

In our study cohort, the degree of PPM was not an independent predictor of survival post AVR or MVR. There was also no significant interaction between LV function and degree of PPM on survival. The risk of aggressive over-sizing of valves and aortic root enlargement procedures especially in an older patient population need to be considered carefully in light of these results as well as previous studies which do not demonstrate survival benefit in patients without PPM. Further studies, potentially using more robust and patient-specific parameters to grade PPM are needed to fully evaluate the impact of PPM on survival as well as exercise capacity and quality of life.

## Data Availability

We agree with availability of data and sharing data.

## Abbreviations

AF: Atrial fibrillation
AR: Aortic regurgitation
AS: Aortic stenosis
AVR: Aortic valve replacement
BMI: Body mass index
BSA: Body surface area
DM: Diabetes mellitus
EF: Ejection fraction
EOA: Effective orifice area
EOAi: Indexed effective orifice area
HR: Hazard ratio
IQR: Inter-quartile range
LV: Left ventricle
LVF: Left ventricular function
MVR: Mitral valve replacement
NYHA: New York Heart Association
PPM: Patient-prosthesis mismatch
PVD: Peripheral vascular disease
RV: Right ventricle
SD: Standard deviation
